# Patients’ Attitudes Toward an Online Patient Portal for Communicating Laboratory Test Results: Real-World Study Using the eHealth Impact Questionnaire

**DOI:** 10.2196/17060

**Published:** 2020-03-04

**Authors:** Esther Talboom-Kamp, Rosian Tossaint-Schoenmakers, Annelijn Goedhart, Anke Versluis, Marise Kasteleyn

**Affiliations:** 1 Saltro Diagnostic Centre Utrecht Netherlands; 2 Public Health and Primary Care Department Leiden University Medical Centre Leiden Netherlands; 3 National eHealth Living Lab Leiden University Medical Centre Leiden Netherlands

**Keywords:** patient portals, eHealth Impact Questionnaire, eHIQ, laboratory test results, attitude to health, self efficacy, telemedicine, usability

## Abstract

**Background:**

Communicating laboratory test results online has several advantages for patients, such as improving clinical efficiency and accessibility, thereby helping patients to take an active role in managing their health.

**Objective:**

This study aimed to investigate the experiences and self-efficacy of patients using an online patient portal that communicates laboratory test results.

**Methods:**

We used the online-administered eHealth Impact Questionnaire to explore patients’ attitudes toward the portal. Patients visiting the portal were asked to complete the questionnaire. The subscale Information and Presentation assessed the usability of the patient portal and the subscale Motivation and Confidence to Act assessed self-efficacy to determine whether patients were motivated to act on the presented information. We used a cutoff score of 65 or greater to determine whether the portal was rated positively.

**Results:**

The questionnaire was completed by 354 of 13,907 patients who viewed their laboratory results in the patient portal, with a response rate of 2.55%. The mean Information and Presentation score was 67.70 (SD 13.12) and the mean Motivation and Confidence to Act score was 63.59 (SD 16.22). We found a positive, significant correlation between the 2 subscales (*r*_345_=.77, *P*<.001).

**Conclusions:**

Patients participating in the study rated the usability of the portal positively. However, the portal only slightly helped patients to take an active role in managing their own health. The low response rate precludes generalization of the results. Future research should examine avenues to further increase patients’ self-efficacy and study whether portal acceptability differs in subgroups. Patient portals conveying laboratory test results in understandable language seem usable and potentially provide a viable way to help patients take a more active role in managing their own health.

## Introduction

### Background

Patient involvement in decision making and delivery of health care is important to patients, health care providers, and policy makers. When patients are activated to be more engaged in health and disease issues, their behavior changes toward more self-management [1]. Therefore, patient involvement is stimulated as an essential element of patient-centered care and as a means to improve the quality and efficiency of care [2,3]. With modern digital possibilities, such as electronic patient portals, patients’ activation and information can be organized more easily. The internet is increasingly being used by care consumers to look for answers about health concerns and has the potential to change health care behavior [4,5]. Although personal health records and patient portals are promising tools, evidence of their effects on patient centeredness of care, efficiency of care, and health outcomes is inconsistent [6-8]. Furthermore, adoption rates of electronic health (eHealth) vary greatly and are often less than 50% [9-13].

Several health care organizations in the Netherlands, such as Saltro diagnostic center, have invested in the development of a high-quality patient portal that is blended into usual care. Solutions that are blended into usual care generally have higher adoption rates [14]. Saltro’s portal provides access to laboratory test results, including explanatory information and visualization, for the individual patient [15]. The aim is to facilitate patients to play an active role in their diagnostic process and disease management. Patient health engagement is indispensable to improve diagnostic accuracy [16]. When patients take an active role in this process, for instance by asking questions and voicing their opinions, it improves the diagnostic process [17]. Consistent with the trend of patients being more proactive and involved in their own health care [18], becoming a more knowledgeable consumer may reduce the risk of diagnostic error [19].

The full potential of patient portals will only be reached if patients understand the results that are communicated, in this case, the information that becomes available from laboratory tests. How the content is presented in a portal and how the patient interprets this affects the overall usefulness of the information [20]. The information in a patient portal can, for example, cause insecurity for the patient—as patients can become emotionally destabilized by the confusion or impact of the test results—which can negatively affect patient health engagement [21]. This risk is more prominent when patients find the results difficult to interpret [22]. Problems have previously been reported with the complexity of the provided information, making it mainly useful for patients with high health literacy [23]. Research has also shown that misinterpreting the risk of blood test outcomes is common, with patients underestimating the severity [24]. These findings raise concerns for patient safety. How results are communicated through patient portals is thus important and needs to be done in a manner that minimizes the risk of misunderstanding. Therefore, testing how patients perceive online portals and test results is recommended, for example, by using the eHealth Impact Questionnaire (eHIQ) [25].

### Objective

Previous research with the Saltro patient portal showed that the presented test results were valuable and important to the majority of the participants (ie, members of a health care consumer panel) [15]. To further scientific knowledge, research is needed to examine how patients perceive the online portal. Therefore, we set up a questionnaire study to explore patients’ attitudes toward a patient portal that was specifically designed to communicate laboratory test results with explanatory texts and supporting visuals. The first aim of this study was to provide insight into the usability of patient portals (including ease of use, perceived trustworthiness, and appropriateness of information). Examining user experience is important, because perceived trustworthiness has been linked to use and engagement with online health information [26,27]. The second aim of this study was to provide insight into how the Saltro laboratory test results portal affects patients’ motivation and confidence to manage their health. This relates to self-efficacy, defined as a person’s confidence in his or her ability to perform specific behaviors that are considered beneficial [28]. Self-efficacy is considered important for motivation and intention to act on information [29]. The third aim of this study was to analyze whether there is a positive association between the perceived usability of the patient portal and self-efficacy, consistent with the literature [30,31]. Overall, this study aimed to assess the experiences and self-efficacy of patients using a patient portal and the association between the 2 constructs.

## Methods

### Design and Participants

We conducted a real-world study between September 2018 and February 2019 to explore patient attitudes toward a patient portal. The participants were patients who received a diagnostic request form from their general practitioner (GP) for a blood test at Saltro, a primary care diagnostic center and laboratory in the Netherlands. Each month approximately 65,000 patients receive a diagnostic request form for a blood test at Saltro. These patients have access to the patient portal, although not all patients use the patient portal. Patients who viewed their test results in the patient portal were approached online to participate in this study by completing an online questionnaire. There were no specific inclusion or exclusion criteria. This study did not require approval from an ethics committee, because no personal information was collected, and the data could therefore not be traced back to the individual.

### Patient Portal

In 2015, Saltro launched a test result Web-based portal that gives patients access to their laboratory test results, including understandable explanatory information personalized to the individual patient (based on sex and age). The portal was created together with health care professionals and patients. All medical content was written by a multidisciplinary team consisting of a GP, a communication specialist, and a clinical chemist. The texts were written to be understandable for the majority of people and have been reviewed by patients and adjusted based on their advice. The level of health literacy of the result information has been estimated at communication level 1B on the scales of the Common European Framework of Reference for Languages [32]. A previous evaluation study showed that over 85% of patients found the accompanying text with the laboratory results comprehensible [33]. Daily, approximately 300 unique individuals look up their laboratory results in the portal. Patients also have the option to share their results with others.

**Figure 1:**
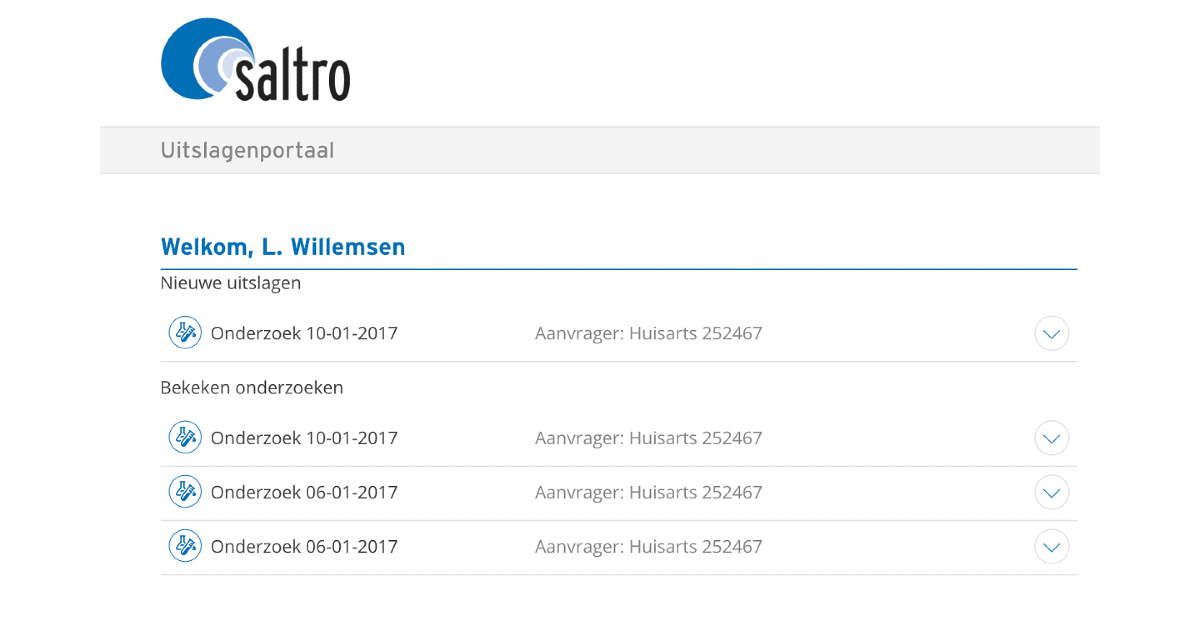
Patient portal overview showing the laboratory tests that were ordered, with the result of the most recent test displayed at the top.

After having blood drawn, patients can look up the test results by logging in to the GP website with a username and password. The login procedure is in line with Dutch security legislation and guidelines (ie, the Dutch Personal Data Protection Act) and the General Data Protection Regulation guidelines. There are no age restrictions to logging in. After logging in, the patient sees an overview of all new and old laboratory tests ordered by date (see Figure 1). This makes it possible to compare new test results with previous results.

**Figure 2:**
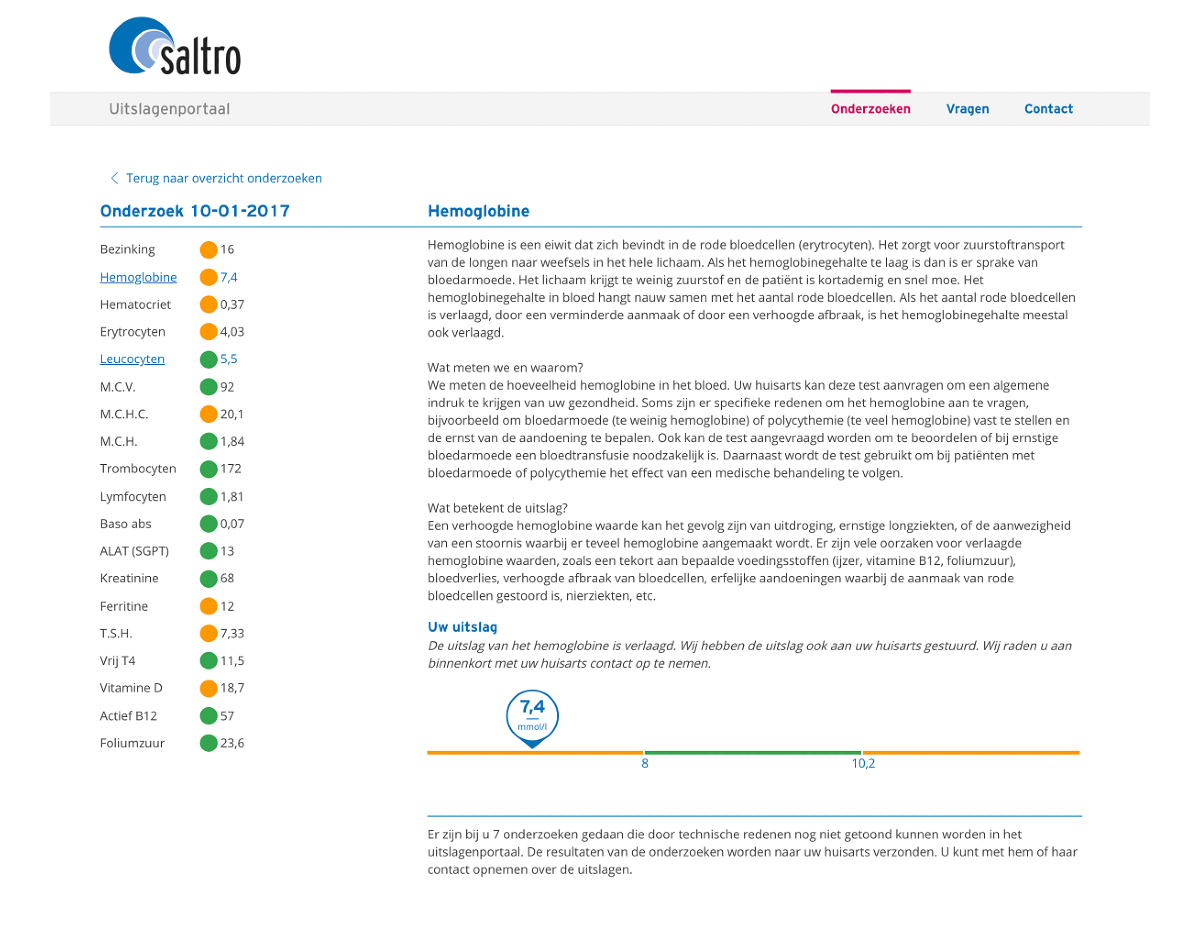
Display of the results of a specific laboratory test in the patient portal.

After clicking on a specific date, the patient is shown the results of the laboratory test that was performed on that date (see Figure 2). For each laboratory test, the patient sees the individual results together with traffic light–colored bullets indicating normal or abnormal results. Clicking on an individual test result shows an explanation of the laboratory test results in a simple and understandable manner. The texts contain an explanation about the test, what was measured, and why a physician might order this test. If a test result is abnormal, then possible diagnoses are mentioned, and patients are advised to discuss the result with the GP. Next, the individual test results are discussed together, and an explanation of what the results could mean for the patient is given.

In addition to the text, a visual is presented underneath the explanatory text (see Figure 2). The visual presents the individual numeric value of the laboratory test result and how it relates to the reference value(s). Colors are added to emphasize this range. The reference values differ per laboratory test, and sometimes also by sex and age. A green dot or line means that the result is normal for the patient, and there is no deviation. An orange dot or line means the laboratory result is divergent or abnormal. As the individual numeric value of the laboratory test is presented above the line, patients can see whether their value is normal or deviates from the reference value. The majority of patients find this information valuable and important [15]. Patients are referred to their GP if they have questions. If the dot or line is red, it means the laboratory result is severely deviating (compared with the reference value). In that case, Saltro directly contacts the GP to get in contact with the patient for suitable treatment. Textbox 1 shows an example of a patient journey.

**Example of a patient journey.**

A person develops complaints about his health and goes to the general practitioner (GP). The GP examines the person and requests blood tests. The person goes to the phlebotomist from Saltro, who collects blood, which is analyzed in the laboratory. The same evening the person can look up the results in the portal. He can see which tests are normal and not likely to be the cause of health complaints. He can see what is tested and will know what is functioning accurately in his body, which will be reassuring. He can also see and choose to read the divergent laboratory results first. He can compare the value with reference values to see how deviating the value is. He does not have to search on the internet; he reads quickly what this test means and can contact the GP to discuss worries and questions, and to make decisions together regarding further steps and treatment.

### Outcome Measure

The primary outcome of this study was the second part of the validated Dutch version of the eHIQ [25,34]. The eHIQ is a self-reported questionnaire of which Part 2 measures patients’ attitudes toward a *specific* health-related website, in this case, the patient portal. We chose the eHIQ for the following reasons. First, the eHIQ assesses the patient’s perspective of the website. Second, the questionnaire is translated and validated in Dutch. Third, information from the eHIQ can be used to compare the effects of the websites for benchmarking; with this study we set a first standard. Fourth, the information can be used to improve a website further, in this case, the patient portal. Each of the 26 items is scored on a 5-point Likert scale ranging from “strongly disagree (1)” to “strongly agree (5).” The questionnaire has 3 subscales: (1) Information and Presentation, (2) Motivation and Confidence to Act, and (3) Identification. The Information and Presentation subscale has 13 items and measures whether people find the website easy to use; this includes items on understanding, trustworthiness, and whether images used were appropriate. This subscale relates to usability. The Motivation and Confidence to Act subscale consists of 10 items and assesses whether an individual felt reassured after reading the information on the website and was motivated to manage their health. This subscale relates to self-efficacy. The final subscale, Identification, consists of 3 items and measures whether individuals identify with others who use the website. An example item is “I feel I have a sense of solidarity with other people using the website.” As users of the patient portal do not interact with other users, we considered this subscale to be irrelevant for this study and therefore did not discuss it further. We transformed the total scores for each subscale to a scale of 0 to 100, with higher scores representing a more positive attitude. No official cutoff score is available to determine whether a website or portal is rated as positive or negative. In consultation with the authors who translated and validated the eHIQ in a Dutch population of eHealth users, we determined that a score of 65 or greater is considered positive. The eHIQ has good construct validity, internal consistency, and test-retest reliability [25,34]. Cronbach alpha in this study was considered good (.88 to .90).

### Procedure

Patients who received a laboratory request form for a blood test at Saltro and who used the patient portal in the period between September 2018 and February 2019 were digitally approached to complete the eHIQ-Part 2. After patients viewed their results in the portal, a pop-up window appeared asking them whether they wanted to fill in a questionnaire. Below this question, the questionnaire was shown to patients and patients could complete it in the portal. Individuals who were unwilling to complete the questionnaire (based on the first question) had to click the pop-up away. These individuals, however, were asked to complete the questionnaire again when they logged in at a later point to view other test results. Patients could complete the questionnaire only once.

Completed questionnaires were automatically sent to us by email. The answers to the questionnaire were coupled to the last test result, indicating whether it was normal or deviant, and the number of laboratory requests for that participant. No personal information of the participant, type of blood test, and the interpretation of the laboratory results were visible to us.

### Statistical Analyses

To gain insight into the patient’s perceived usability of the patient portal and their self-efficacy of using a patient portal, we performed descriptive statistics. We calculated the mean scores of the 2 eHIQ subscales and used a cutoff score of 65 or greater to determine how the portal was rated. When the mean of the subscale was 65 or higher, we evaluated the subscale positively. Also, we examined the highest- and lowest-scoring items for each subscale to get a better understanding of which aspects of the patient portal were appreciated and which could be improved further. For items with the same mean score, we chose the items with the highest precision. To examine whether the perceived usability of the patient portal (first subscale, Information and Presentation) was positively associated with self-efficacy (second subscale, Motivation and Confidence to Act), we performed a Pearson correlation. Data were normally distributed and we identified no significant outliers. We performed all analyses using IBM SPSS Statistics version 24 (IBM Corporation).

## Results

A total of 13,907 patients viewed their laboratory results on the patient portal and were invited to complete the eHIQ. The questionnaire was completed by 354 patients (2.55%). These participants completed all items of the eHIQ. The mean score of the subscale Information and Presentation was 67.70 (13.12) on a scale ranging from 0 to 100. This subscale of eHIQ thus scored above the set cutoff score of 65 and was evaluated positively. The mean score of the subscale Motivation and Confidence to Act was 63.59 (SD 16.22) on a scale of 0 to 100. This score was just below the set cutoff score and was therefore not considered positively evaluated. Table 1 presents the mean scores of the 2 subscales and the individual items.

We identified the 3 highest- and lowest-scoring items of the 2 subscales. The highest-scoring items on Information and Presentation were trust in the provided information (mean 4.06, SD 0.69), ease of understanding the information (mean 4.06, SD 0.81), and use of understandable language in the portal (mean 4.04, SD 0.80). The lowest-scoring items were about whether the images were distressing (mean 3.44, SD 0.79), tips were useful (mean 3.27, SD 0.94), and website imagery was appropriate (mean 3.27, SD 0.71). The highest-scoring items on Motivation and Confidence to Act were on better understanding personal health by using the website (mean 3.86, SD 0.74), being encouraged to take health-beneficial actions (mean 3.85, SD 0.93), and confidence to take action (mean 3.56, SD 0.84). The lowest-scoring items were on whether the website would be consulted to make a decision about health (mean 3.38, 0.95), gives confidence to discuss health with other people (mean 3.37, SD 0.94), and gives confidence to explain health concerns to others (mean 3.36, SD 0.91).

To examine whether the perceived usability of the patient portal was positively associated with self-efficacy, we calculated a Pearson correlation. There was a large, positive, significant correlation between the subscale Information and Presentation and Motivation and Confidence to Act (*r*_345_=.77,*P*P<.001). This finding was in line with our expectations.

**Table 1:** Mean scores of the 2 subscales of the eHealth Impact Questionnaire (eHIQ)-Part 2 and the individual itemsa.

Subscale and item		Score, mean (SD)
**Information and Presentation**		67.70 (13.12)
	I trust the information on the website	4.06 (0.69)
	I can easily understand the information on the website	4.06 (0.81)
	The language on the website made it easy to understand	4.04 (0.80)
	The information on the website left me feeling confused^b^	3.95 (0.98)
	I value the advice given on the website	3.79 (0.78)
	The website is easy to use	3.82 (0.89)
	The website provides a wide range of information	3.73 (0.83)
	The website has a positive outlook	3.64 (0.88)
	The people who have contributed to the website understand what is important to me	3.63 (0.79)
	On the whole, I find the website reassuring	3.51 (0.82)
	I found the images on the website distressing^b^	3.44 (0.79)
	The website includes useful tips on how to make life better	3.27 (0.94)
	Photographs and other images were used appropriately on the website	3.27 (0.71)
**Motivation and Confidence to Act**		63.59 (16.22)
	The website helps me to have a better understanding of my personal health	3.86 (0.74)
	The website encourages me to take actions that could be beneficial to my health	3.85 (0.93)
	The website gives me confidence that I am able to manage my health	3.56 (0.84)
	The website encourages me to play a more active role in my health care	3.56 (0.88)
	I have learned something new from the website	3.55 (0.97)
	I feel more inclined to look after myself after visiting the website	3.53 (0.87)
	The website prepares me for what might happen to my health	3.42 (0.91)
	I would consult the website if I had to make a decision about my health	3.38 (0.95)
	The website makes me more confident to discuss my health with the people around me (for example, my family, or people at work)	3.37 (0.94)
	The website gives me the confidence to explain my health concerns to others	3.36 (0.91)

^a^Although the Dutch version of the eHIQ was used in this study, for the purpose of this paper the items from the standard English-language version of the eHIQ are shown.

^b^This item was reverse scored.

## Discussion

### Principal Findings

This study aimed to investigate patients’ attitudes toward a patient portal specifically designed to communicate laboratory test results, thereby helping patients to take an active role in managing their own health. Findings showed that the usability of the patient portal, assessed by the subscale Information and Presentation of the eHIQ, was rated positively. This suggests that study participants found the patient portal easy to use, considered it trustworthy and appropriate, and found the provided information easy to understand. The self-efficacy of patients using the patient portal, indicative of patients’ motivation and confidence to act on the presented information, also received a relatively high score, but this score was just below the set cutoff score that we used to determine whether patients’ attitudes toward the portal were positive. In addition, as expected, we found a positive association between the portal’s usability and patients’ self-efficacy [30,31]. Altogether, the findings show that patients were generally positive toward the portal, but it is important to identify opportunities to further optimize patients’ self-efficacy, as this affects a person’s intention to act on the information.

### Comparison With Prior Work

The usability of the patient portal, which includes patient understanding, was rated positively. This is important because, if all patients are to receive their test results automatically online, the portal needs to be easy to use and provide information that is understandable for all. The high score on usability is in line with previous research examining patient portals with laboratory test results [35-37]. The lowest-scoring items on usability were on provided tips and imagery, which we considered less relevant for this patient portal, as the portal does not include tips or imagery. Therefore, the actual usability of this particular patient portal might have been higher than this study found it to be. As no sociodemographic information was available, we could not determine whether the results differed by subgroup (eg, age, sex, level of health literacy). Future studies should examine whether the patient portal with laboratory test results is usable for all.

As mentioned above, the self-efficacy of patients using the portal—measured with the Motivation and Confidence subscale—was slightly lower than the set cutoff score. Considering that this was, to our knowledge, the first study of a patient portal to use the eHIQ, no official cutoff was available, and this limits our ability to compare this study’s self-efficacy score with other studies’ results. Moreover, to the best of our knowledge, no studies have examined patients’ self-efficacy with questionnaires other than the eHIQ after being presented with online laboratory test results. Both usability and self-efficacy affect an individual’s intention to follow up the test result [29,38]. Therefore, it is important that these factors be evaluated and improved where needed. We discuss some potential avenues for improvement below.

One potential area to improve is the use of reference values when communicating laboratory test results. Currently, a visual presents how the numeric value of the laboratory test result relates to a reference value that takes sex and age into account (when relevant). This standard reference value might, however, be less relevant for individuals with a chronic condition (eg, diabetes). Research has now shown that using reference values that are clinically appropriate (ie, personalized) can help to improve patients’ understanding and decrease negative responses to the results [39]. Replacing standard reference values with clinically relevant values will not be relevant for all laboratory tests (eg, not for sexually transmitted infection tests), but might be useful for other tests (eg, glucose, kidney function), and future studies should investigate this possibility.

A second potential area to improve is the understanding and effective use of laboratory test results by providing additional information [40]. One study showed that 50% of patients using a portal accessed additional, external information related to the diagnostics test results [36]. Adding additional information, however, might also increase the complexity of the presented information and this, in turn, might decrease understanding and limit a patient’s ability to extract the relevant information [41]. This highlights the need to find the right balance between providing enough information and information overload. Adding links to additional information might provide a solution, by making more in-depth information easily available to those interested, while not running the risk of overwhelming patients with large volumes of text.

A third potential area to improve relates to patient portal use being predicted by perceived usefulness and perceived ease of use [38]. This emphasizes the necessity to involve end users when designing patient portals to ensure that the portal is perceived as useful and easy to use [42]. The Saltro patient portal was developed in close collaboration with both patients and health care providers, thereby attempting to address the end users’ needs and assure usability. Nevertheless, it is important to continually evaluate these aspects to ensure that they are adequately met and to identify areas for future improvements.

### Limitations and Strengths

Even though communicating laboratory test results online can have some advantages, such as improving clinical efficiency and improving accessibility of results, there is a limited number of studies on the use of such systems [41,43]. This study, therefore, adds to the limited existing literature base. Some limitations, however, also need to be discussed. First, the response rate was low and, consequently, there is risk of self-selection bias. A low response rate, however, does not automatically equal low study quality, as a low response rate is only problematic when it affects the sample’s representativeness [44]. Still, 97.45% (13,553/13,907) of the patients did not complete the study questionnaire. This high rate of noncompletion precludes generalizing whether the patient portal display and explanation of results are acceptable and informative for all patients.

Second, as mentioned above, no sociodemographic information was available from participants. This restricted us from doing subgroup analyses to see whether attitudes regarding the portal were dependent on these characteristics. Limited research is available on whether portal use and acceptance differ between groups. One study did find that portal use was influenced by age, presence of a chronic illness, and eHealth literacy level [39]. Further research into potential group differences is necessary, and such information can be used to fine-tune the portal to make it acceptable for every user.

Third, in some cases, it is important that patients act on the test results presented in the portal. Even though self-efficacy can be a valuable predictor of action [45], it is still a proxy of action and it would be interesting to study the effect on actual behavioral activation.

A strength of this study is that patients completed the questionnaire immediately after they accessed the portal and viewed their results, thereby limiting recall bias and giving an accurate picture of patients’ attitudes toward the portal.

### Conclusions

Study participants evaluated the usability of Saltro’s online patient portal communicating laboratory test results positively. Nevertheless, it should be noted that the low response rate precludes generalization of the results. Patients’ motivation and confidence to act on the presented information also scored relatively high, but future research should examine ways to further optimize patients’ self-efficacy to increase an individual’s intention to act on the information. In addition, it is important to determine potential group differences in portal use and acceptance. Overall, study participants had a positive attitude toward the patient portal and the portal potentially can help patients take a more active role in managing their own health.

## Abbreviations

eHealth: electronic health
eHIQ: eHealth Impact Questionnaire
GP: general practitioner
